# Validation of genome-wide association study-identified single nucleotide polymorphisms in a case-control study of pancreatic cancer from Taiwan

**DOI:** 10.1186/s12929-020-00664-9

**Published:** 2020-05-26

**Authors:** Yan-Shen Shan, Li-Tzong Chen, Jin-Shang Wu, Yin-Fan Chang, Chih-Ting Lee, Chih-Hsing Wu, Nai-Jung Chiang, Hsin-En Huang, Chia-Jui Yen, Ying-Jui Chao, Hui-Jen Tsai, Chiung-Yu Chen, Jui-Wen Kang, Chin-Fu Kuo, Chia-Rung Tsai, Ya-Ling Weng, Han-Chien Yang, Hui-Chin Liu, Jeffrey S. Chang

**Affiliations:** 1 Department of Surgery, National Cheng Kung University Hospital, National Cheng Kung University, 138 Sheng Li Road, Tainan, 70456 Taiwan; 2 Institute of Clinical Medicine, College of Medicine, National Cheng Kung University, Tainan, 138 Sheng Li Road, Tainan, 70456 Taiwan; 3 National Institute of Cancer Research, National Health Research Institutes, 1F No 367, Sheng-Li Road, Tainan, 70456 Taiwan; 4 Department of Internal Medicine, National Cheng Kung University Hospital, National Cheng Kung University, 138 Sheng Li Road, Tainan, 70456 Taiwan; 5 Department of Internal Medicine, Kaohsiung Medical University Hospital, Kaohsiung Medical University, Ziyou 1st Road, Sanmin District, Kaohsiung, 80756 Taiwan; 6 Institute of Molecular Medicine, College of Medicine, National Cheng Kung University, 138 Sheng Li Road, Tainan, 70456 Taiwan; 7 Department of Family Medicine, National Cheng Kung University Hospital, National Cheng Kung University, 138 Sheng Li Road, Tainan, 70456 Taiwan; 8 Preventive Medicine Center, Taichung Tzu Chi Hospital, 88 Section 1, Fengxing Road, Tanzi District, Taichung, 427 Taiwan; 9 Department of Nursing, National Cheng Kung University Hospital, National Cheng Kung University, 138 Sheng Li Road, Tainan, 70456 Taiwan

**Keywords:** Genome-wide association, Epidemiology and prevention, Pancreatic cancer, Gene-environment interaction

## Abstract

**Background:**

Due to differences in genetic background, it is unclear whether the genetic loci identified by the previous genome-wide association studies (GWAS) of pancreatic cancer also play significant roles in the development of pancreatic cancer among the Taiwanese population.

**Methods:**

This study aimed to validate the 25 pancreatic cancer GWAS-identified single nucleotide polymorphisms (SNPs) in a case-control study (278 cases and 658 controls) of pancreatic cancer conducted in Taiwan. Statistical analyses were conducted to determine the associations between the GWAS-identified SNPs and pancreatic cancer risk. Gene-environment interaction analysis was conducted to evaluate the interactions between SNPs and environmental factors on pancreatic cancer risk.

**Results:**

Among the 25 GWAS-identified SNPs, 7 (rs2816938 (~ 11 kb upstream of *NR5A2*), rs10094872 (~ 28 kb upstream of *MYC*), rs9581943 (200 bp upstream of *PDX1*) and 4 chromosome 13q22.1 SNPs: rs4885093, rs9573163, rs9543325, rs9573166) showed a statistically significant association with pancreatic cancer risk in the current study. Additional analyses showed two significant gene-environment interactions (between poor oral hygiene and *NR5A2* rs2816938 and between obesity and *PDX1* rs9581943) on the risk of pancreatic cancer.

**Conclusions:**

The current study confirmed the associations between 7 of the 25 GWAS-identified SNPs and pancreatic risk among the Taiwanese population.

Furthermore, pancreatic cancer was jointly influenced by lifestyle and medical factors, genetic polymorphisms, and gene-environment interaction. Additional GWAS is needed to determine the genetic polymorphisms that are more relevant to the pancreatic cancer cases occurring in Taiwan.

## Introduction

According to the International Agency for Research on Cancer (IARC), approximately 458,918 cases (incidence rate = 4.8 per 100,000) of pancreatic cancer are diagnosed worldwide annually [1]. The survival of pancreatic cancer is extremely poor with a 5-year survival rate of only 9% [2], resulting in the high number of deaths (approximately 432,242 deaths per year) that almost equals the number of incident cases [1].

Despite the numerous epidemiologic investigations of pancreatic cancer, the only established environmental risk factors (defined as factors other than genetic factors such genetic polymorphisms or mutations) with strong evidence are cigarette smoking and chronic diabetes [3, 4]. Environmental factors that have shown a strong association with an increased pancreatic cancer risk include obesity and chronic pancreatitis [5–7], while allergies have been consistently associated with a decreased pancreatic cancer risk [8]. Other environmental factors that may possibly influence the risk of pancreatic cancer but require more investigations are diet, physical exercise, oral hygiene/health, and alcohol use. Diet rich in fruits and vegetables has been associated with a reduced pancreatic cancer risk while diet with higher levels of meat and animal products has been linked to an increased pancreatic cancer risk [9–11]. A reduced risk of pancreatic cancer has been linked to higher levels of physical activities, although results have not been consistent across studies [12–15]. Huang et al. used the number of teeth, dental plaque, and oral mucosal lesions as indicators of oral hygiene status and reported that lower number of teeth, higher level of dental plaque, and presence of oral mucosal lesions were associated with a higher risk of pancreatic cancer [16]. For alcohol drinking, studies suggested that only high amount of alcohol consumption may increase the risk of pancreatic cancer [14, 17, 18].

Only five to 10 % of pancreatic cancers are familial with strong genetic predisposition due to rare genetic mutations [19, 20]. The majority of the pancreatic cancer cases are sporadic with an unclear level of genetic contribution. Numerous candidate gene studies that selected genetic polymorphisms based on a priori hypothesis have reported genetic polymorphisms associated with pancreatic cancer risk, including polymorphisms of DNA repair genes, carcinogen-metabolizing genes, folate-metabolizing genes, and alcohol-metabolizing gene. These studies were documented by a systematic review and meta-analysis by Dai et al. [21]. According to their study, many of the polymorphisms were investigated by only 1 or 2 studies and therefore the reliability of the results required further validation. They performed meta-analysis on polymorphisms that had been investigated by three or more studies, which focused mainly on the DNA repair and folate-metabolizing genes. Their analysis found that polymorphisms of the DNA repairs genes, including *XRCC1* Arg399Gln and Arg194Trp, *ERCC1* rs11615 and rs3212986, and *ERCC2* rs13181, showed significant association with pancreatic cancer risk. No significant association was found between the C677T and A1298C polymorphisms of the folate-metabolizing gene, *MTHFR,* and pancreatic cancer risk [21]. Because most candidate gene studies were conducted with a relatively small sample size, the results across studies were often inconsistent and not replicable.

In contrast to candidate gene studies, genome-wide association studies (GWAS) are usually performed with a large sample size consisting of discovery and replication groups and therefore the results of GWAS are usually considered more valid than those generated by the candidate gene studies. GWAS have identified genetic polymorphisms that are associated with the risk of pancreatic cancer [22–33]. These studies, conducted among different racial/ethnic groups, have identified genetic loci that are common across and unique to different racial/ethnic groups. Due to differences in genetic background, it is unclear whether these genetic loci identified by the previous GWAS of pancreatic cancer also play significant roles in the development of pancreatic cancer among the Taiwanese population. The current study aimed to validate the pancreatic cancer GWAS-identified single nucleotide polymorphisms (SNPs) in a case-control study of pancreatic cancer conducted in Taiwan.

Previous studies have reported significant gene-environment interactions on the risk of pancreatic cancer. For example, the increased pancreatic cancer risk due to cigarette smoking was modified by polymorphisms of carcinogen-metabolizing, folate-metabolizing, and DNA repair genes [34–36]. An interaction between diabetes and the polymorphisms of hexokinase 2 (*HK2*), a glucose metabolism gene, on the risk of pancreatic cancer was reported, with *HK2* R844K GA/AA genotype showing an inverse association with pancreatic cancer among individuals without diabetes, but a positive association with pancreatic cancer among diabetic patients [37]. These studies indicated that pancreatic cancer could result from the interplay between environmental and genetic factors; therefore, the current study also performed exploratory analysis to evaluate the interaction between the GWAS-identified SNPs and the environmental factors on the risk of pancreatic cancer.

## Materials and methods

The current study was approved by institutional review boards of the National Cheng Kung University Hospital (ethic approval number: B-BR-102-070) and the National Health Research Institutes (ethic approval number: EC1030109-E). A signed informed consent was collected from every study participant who agreed to participate in the study.

### Subject recruitment

All subjects were from an ongoing case-control study of pancreatic cancer conducted at the National Cheng Kung University Hospital. Cases were recruited from the Department of General Surgery or the Division of Hemato-Oncology, Department of Internal Medicine. The eligible criteria for the cases were: 1) diagnosis of pancreatic ductal adenocarcinoma 2) no history of previous cancer diagnosis; 3) age = 20 years or more; and 4) the ability to understand the purpose of the study and provide informed consent. Control subjects were recruited from the Department of Family Medicine and the eligibility criteria were: 1) the purpose of the clinical visit was for regular physical examination, vaccination, or for minor illnesses (e.g. common cold, headache) not related to cigarette smoking or metabolic diseases (diabetes, hypertension, hyperlipidemia); 2) no history of previous cancer diagnosis; 3) age = 20 years or more; and 4) the ability to understand the purpose of the study and provide informed consent. The current analysis included subjects recruited from November 19, 2013 to February 7, 2018.

### In-person interview

Each study subject was interviewed in-person by a trained interviewer using a standardized questionnaire to collect information on: 1) demographic information, including age, sex, and education; 2) lifestyle factors, including cigarette smoking, alcohol drinking, and diet; 3) medical history, including history of allergy and diabetes/glucose intolerance; 4) oral hygiene habits, including regular dental visits, frequency of tooth brushing, and use of dental floss; and 5) biometric measurements, including height and weight 2 years before the pancreatic cancer diagnosis for the cases or before the interview date for the controls. We asked about the weight 2 years before the diagnosis for the cases because we wanted to collect the weight information before the development of pancreatic cancer to avoid reverse causation.

### DNA sample collection and processing

DNA samples were obtained from blood or buccal swab for those who could not provide blood samples. Blood samples were collected in a vacutainer tube containing EDTA (lavender-top). Buccal swab samples were obtained by gently brushing the buccal mucosa with FLOQSwabs (Copan Flock Technologies, Brescia, Italy). Blood samples were centrifuged to separate out the buffy coat. Genomic DNA was extracted from the buffy coat and the buccal swab samples using a commercially available DNA purification kit. DNA samples were stored in the − 80 °C refrigerator until ready to use.

### SNP selection

Literature search was conducted to identify pancreatic cancer GWAS articles and related articles published by December 31, 2017. A total of 12 articles were identified (Supplementary Table 1). Eight of the 12 articles included all or mostly subjects with European ancestry and 4 articles included only Asian study subjects (two were Chinese studies and two were Japanese studies). SNPs that showed statistically significant association with pancreatic cancer and were successfully validated in another independent group were identified from these articles. The minor allele frequencies of these SNPs in the East Asian population recorded by the NCBI dbSNP database (https://www.ncbi.nlm.nih.gov/snp/) were reviewed. SNPs with a minor allele frequency < 0.05 were excluded. Twenty-five SNPs were selected for genotyping for the current study. Detailed information for these 25 SNPs can be found in Supplementary Table 1.

### Genotyping

Genotyping of the 25 SNPs was performed using the MassArray System (Agena Bioscience, San Diego, CA, USA), which is a mass spectrometry-based detection system for medium-throughput genotyping. All of the 25 SNPs had a call rate of more than 98%. Samples with a call rate of < 90% were excluded from the analysis.

### Statistical analysis

The average age was compared between cases and controls using t-test. The distributions of sex and education levels were compared between cases and controls using chi-squared test. For analysis with the environmental (non-genetic) factors, we only analyzed factors with strong evidence for association with pancreatic cancer according to the current literature [38], including cigarette smoking, oral hygiene, consumption of vegetables and fruits, allergy, diabetes/glucose intolerance, and body mass index (BMI). First, unconditional logistic regression analysis was conducted to estimate the odds ratio (OR) and 95% confidence interval (CI) to determine the association between each environmental factor and the risk of pancreatic cancer, adjusted for sex, age, and education. Subsequently, another model including all of the above-mentioned environmental factors plus adjustment for sex, age, and education was constructed to assess the independent contribution of each environmental factor while adjusting for the potential confounding effect of the other environmental factors. Cigarette smoking was analyzed as ever (current + former) vs. never. An oral hygiene score was created to evaluate oral hygiene. The oral hygiene score = regular dental visit + frequency of tooth brushing + use of dental floss: regular dental visit: yes = 0, no = 1; frequency of tooth brushing: ≧ 2 times per day = 0, < 2 times per day = 1; and use of dental floss: yes = 0, no = 1. A higher oral hygiene score indicates poorer oral hygiene. We have used this oral hygiene score previously in several studies of head and neck cancer [39–41]. Furthermore, we have shown that a higher oral hygiene score was correlated with a higher percentage of periodontopathogenic bacteria in the saliva, thus validating the use of the oral hygiene score as an indicator of oral hygiene status [40]. Consumption of vegetables and fruits was evaluated according to the portion and the frequency of intake. Diabetes/glucose intolerance was evaluated according to the time of onset with < 2 years being recent onset and ≧ 2 years being chronic. BMI was calculated with the formula: (weight in kilograms)/(height in meters)^2^. BMI was divided into four categories according to the guideline set by the Ministry of Health and Welfare, Taiwan: < 18.5 = underweight; 18.5–23.9: normal; 24–26.9 = overweight; and 27 or more = obese.

Unconditional logistic regression analysis was conducted to estimate the OR and 95% CI for the association between each SNP and the risk of pancreatic cancer, adjusted for sex, age, and education. SNPs with a *p* < 0.05 were considered statistically significant. Linkage disequilibrium (LD) analysis was performed to identify the statistically significant SNPs in high LD (*r*^*2*^ > 0.8) and the SNP with the lowest p among the highly-linked SNPs was chosen to perform further analyses.

Exploratory gene-environment interaction analysis was performed to determine whether the association between each of the environmental factors and pancreatic cancer risk might differ by the genotype of the SNPs significantly associated with pancreatic cancer. To evaluate the significance of the gene-environment interaction, an interaction term (environmental factor x SNP) was included in the logistic regression model. Log-likelihood ratio test was used to compare the logistic regression model with the interaction term to the model without the interaction term. A *p* < 0.05 for the interaction term was considered statistically significant.

Receiver Operating Characteristic (ROC) curve analysis was conducted to evaluate the capability of the environmental factors and SNPs in differentiating between pancreatic cancer cases and controls. An ROC curve was generated from each of the four risk models constructed using logistic regression: Model 1 included age, sex, education, and environmental factors significantly associated with pancreatic cancer; Model 2 included age, sex, education, and the unlinked SNPs significantly associated with pancreatic cancer. Model 3 included age, sex, education, environmental factors, and SNPs; and Model 4 included age, sex, education, environmental factors, SNPs, and interactions between environmental factors and SNPs. The c-statistic was used to calculate the area under the curve (AUC) to evaluate how well each of the models could differentiate between cases and controls. The AUCs of the different models were compared using the nonparametric approach proposed by Delong et al. [42].

## Results

The current study initially included 285 pancreatic cancer cases and 664 controls, with a participation rate of 86% for the cases and 81% for the controls. Thirteen subjects (7 cases and 6 controls) were excluded from the analysis due to a low sample call rate (< 90%) for the 25 SNPs. The final analysis included 278 pancreatic cancer cases and 658 controls. The majority (86%) of the cases were interviewed within 6 months of the pancreatic cancer diagnosis (76% were interviewed within 3 months and 10% were interviewed between 3 and 6 months). Due to the ongoing study subject recruitment, the age and the sex distributions were imbalanced despite the implementation of frequency matching. Cases were older in mean age and had a lower percentage of women compared to the controls (Table 1). Compared to the cases, a higher percentage of the controls completed at least a college education.

**Table 1:** Demographic characteristics of the pancreatic cancer patients and control subjects

Characteristics	Cases ***N*** = 278 n (%)	Controls ***N*** = 658 n (%)	***P***-value^a^
**Age (years)**			
Mean (SE)	62.1 (0.4)	58.4 (0.6)	< 0.0001
**Sex**			
Men	164 (59.0)	282 (42.9)	< 0.0001
Women	114 (41.0)	376 (57.1)	
**Education**			
≦ Junior high	134 (48.2)	191 (29.0)	< 0.0001
High school/technical school	84 (30.2)	178 (27.1)	
College	50 (18.0)	229 (34.8)	
Graduate school	10 (3.6)	60 (9.1)	

*Abbreviations*: *N* number, *SE* standard error

^a^*P*-values were generated using T-tests (for continuous variables) or chi-squared tests (for categorical variables)

In the logistic regression adjusted only for age, sex, and education (analysis 1), cigarette smoking, poor oral hygiene (i.e. higher oral hygiene score), diabetes/glucose intolerance, and BMI ≧ 27 were all associated with an increased pancreatic cancer risk (Table 2). History of allergy and more frequent consumption of vegetables (> 3 portions per week) and fruits (> 1 portion per day) were associated with a reduced pancreatic cancer risk. In analysis 2, a multivariate logistic regression model containing all of the above-mentioned environmental factors showed that all except for consumption of fruits remained significantly associated with pancreatic cancer risk.

**Table 2:** The association between lifestyle and clinical factors and pancreatic cancer risk

Characteristics	Cases ***N*** = 278 n (%)	Controls ***N*** = 658 n (%)	Analysis 1 OR (95% CI)^a^	Analysis 2 OR (95% CI)^b^
**Cigarette smoking**				
Never	162 (58.3)	496 (75.4)	Reference	Reference
Ever	116 (41.7)	162 (24.6)	1.63 (1.10–2.40)	1.61 (1.05–2.46)
**Oral hygiene score**^c^				
0 (Good)	27 (9.7)	180 (27.4)	Reference	Reference
1	65 (23.4)	219 (33.3)	1.63 (0.98–2.70)	1.44 (0.85–2.43)
2	118 (42.4)	198 (30.1)	2.88 (1.77–4.69)	2.15 (1.28–3.62)
3 (Poor)	60 (21.6)	52 (7.9)	4.60 (2.56–8.29)	2.58 (1.37–4.86)
Unknown	8 (2.9)	9 (1.4)	–	–
**Vegetable consumption**				
3 portions or less per week	106 (38.1)	100 (15.2)	Reference	Reference
> 3 portions per week	172 (61.9)	558 (84.8)	0.29 (0.21–0.41)	0.34 (0.24–0.49)
**Fruit consumption**				
1 portion or less per day	85 (30.6)	143 (21.7)	Reference	Reference
> 1 portion per day	193 (69.4)	513 (78.0)	0.71 (0.51–1.00)	0.95 (0.80–1.13)
Unknown	0 (0.0)	2 (0.3)	–	–
**Allergy**				
No	227 (81.6)	405 (61.6)	Reference	Reference
Yes	51 (18.4)	251 (38.1)	0.46 (0.32–0.65)	0.47 (0.32–0.69)
Unknown	0 (0.0)	2 (0.3)	–	–
**Diabetes mellitus/glucose intolerance (DM/GI)**				
No DM/GI	196 (70.5)	602 (91.5)	Reference	Reference
< 2 years	22 (7.9)	16 (2.4)	4.08 (2.01–8.25)	3.04 (1.42–6.50)
> 2 years	59 (21.2)	39 (5.9)	3.71 (2.36–5.87)	2.84 (1.74–4.61)
Unknown	1 (0.4)	1 (0.2)	–	–
**BMI 2 years ago**^d^				
< 18.5	4 (1.4)	36 (5.5)	0.37 (0.12–1.09)	0.38 (0.12–1.17)
18.5–23.9	102 (36.7)	319 (48.5)	Reference	Reference
24–26.9	83 (29.9)	191 (29.0)	1.07 (0.75–1.53)	0.99 (0.67–1.47)
27 or more	87 (31.3)	111 (16.9)	2.09 (1.43–3.06)	1.68 (1.11–2.54)
Unknown	2 (0.7)	1 (0.1)	–	–

*Abbreviations*: *BMI* body mass index, *CI* confidence interval, *DM/GI* diabetes mellitus/glucose intolerance, *OR* odds ratio

^a^OR and 95% CI of each independent variable in Model 1 was calculated using unconditional logistic regression, adjusted for sex, age, and education

^b^Model 2 included all of the independent variables in the same model with additional adjustment for sex, age, and education

^c^Oral hygiene score = tooth brushing + use of dental floss + regular dental visit, with tooth brushing: ≧2 times per day = 0, < 2 times per day = 1; use of dental floss: yes = 0, no = 1; and regular dental visit: yes = 0, no = 1

^d^BMI at two years before the pancreatic cancer diagnosis for the cases or before the interview date for the controls

Among the 25 pancreatic cancer GWAS SNPs, 7 (rs2816938 (~ 11 kb upstream of *NR5A2*), rs10094872 (~ 28 kb upstream of *MYC*), rs9581943 (200 bp upstream of *PDX1*), and 4 chromosome 13q22.1 SNPs: rs4885093, rs9573163, rs9543325, rs9573166) showed statistically significant association with pancreatic cancer risk (Table 3 and the results for the entire 25 SNPs can be found in Supplementary Table 2). For these 7 SNPs, having at least one copy of the minor allele (dominant inheritance model) was associated with an increased pancreatic cancer risk ranging from 1.4 to 1.7 times. The 4 chromosome 13q22.1 SNPs, rs4885093, rs9573163, rs9543325, and rs9573166, are in high LD (*r*^*2*^ > 0.93); therefore, out of these 4 linked SNPs, we chose the SNP with the lowest p (rs4885093) for further analysis.

**Table 3:** Results for the significant associations between 7 genome-wide association study-identified single nucleotide polymorphisms and the risk of pancreatic cancer

SNPs (chromosome locus, nearest gene (s))	Cases n (%)	Controls n (%)	OR (95% CI)^a^	P
**rs2816938 (**1q32.1, *NR5A2*)				
TT	232 (83.5)	583 (88.7)	Reference	
AT	44 (15.8)	72 (11.0)	1.68 (1.09–2.58)	0.02
AA	2 (0.7)	2 (0.3)	1.58 (0.22–11.39)	0.65
AT+AA	46 (16.6)	74 (11.3)	1.68 (1.10–2.55)	0.02
Every 1 copy of A			1.61 (1.08–2.40)	0.02
**rs10094872** (8q24.21, *MYC*)				
AA	159 (57.2)	429 (65.3)	Reference	
TA	111 (39.9)	196 (29.8)	1.61 (1.19–2.22)	0.002
TT	8 (2.9)	32 (4.9)	0.62 (0.27–1.44)	0.27
TA + TT	119 (42.8)	228 (34.7)	1.48 (1.09–2.00)	0.01
Every 1 copy of T			1.23 (0.95–1.59)	0.11
**rs9581943** (13q12.2, *PDX1*)				
GG	111 (40.1)	323 (49.1)	Reference	
GA	126 (45.5)	250 (38.0)	1.41 (1.03–1.94)	0.03
AA	40 (14.4)	85 (12.9)	1.38 (0.87–2.17)	0.17
GA + AA	166 (59.9)	335 (50.9)	1.40 (1.04–1.89)	0.03
Every 1 copy of A			1.23 (1.00–1.52)	0.05
**rs4885093** (13q22.1, intergenic region)				
AA	59 (21.2)	196 (29.8)	Reference	
AG	150 (54.0)	335 (50.9)	1.43 (0.99–2.06)	0.06
GG	69 (24.8)	127 (19.3)	1.65 (1.07–2.54)	0.02
AG + GG	219 (78.8)	462 (70.2)	1.49 (1.05–2.11)	0.02
Every 1 copy of G			1.29 (1.04–1.59)	0.02
**rs9573163** (13q22.1, intergenic region)				
GG	59 (21.2)	195 (29.6)	Reference	
GC	150 (54.0)	337 (51.2)	1.40 (0.97–2.02)	0.07
CC	69 (24.8)	126 (19.2)	1.64 (1.06–2.53)	0.03
GC + CC	219 (78.8)	463 (70.4)	1.47 (1.04–2.08)	0.03
Every 1 copy of C			1.28 (1.03–1.59)	0.02
**rs9543325** (13q22.1, intergenic region)				
TT	62 (22.3)	198 (30.1)	Reference	
TC	146 (52.5)	333 (50.7)	1.40 (0.99–1.98)	0.06
CC	70 (25.2)	126 (19.2)	1.77 (1.18–2.67)	0.006
TC + CC	216 (77.7)	459 (69.9)	1.44 (1.02–2.02)	0.04
Every 1 copy of C			1.28 (1.03–1.58)	0.02
**rs9573166** (13q22.1, intergenic region)				
AA	62 (22.4)	193 (29.4)	Reference	
GA	145 (52.3)	335 (51.0)	1.29 (0.90–1.86)	0.17
GG	70 (25.3)	129 (19.6)	1.56 (1.02–2.39)	0.04
GA + GG	215 (77.6)	464 (70.6)	1.37 (0.97–1.93)	0.08
Every 1 copy of G			1.25 (1.01–1.55)	0.04

*Abbreviations*: *CI* confidence interval, *OR* odds ratio

^a^OR and 95% CI were calculated using unconditional logistic regression, adjusted for age, sex, and education

We conducted a multivariate analysis that included the four unlinked SNPs (*NR5A2* rs2816938, *MYC* rs10094872, *PDX1* rs9581943 and a chromosome 13q22 SNP, rs4885093). The results of the multivariate analysis showed that each of the four SNPs was independently associated with the risk of pancreatic cancer (Table 4).

**Table 4:** Multivariate analysis to assess the independent association between the four unlinked single nucleotide polymorphisms and pancreatic cancer risk

SNPs (chromosome locus, nearest gene (s))	Cases n (%)	Controls n (%)	OR (95% CI)^a^	P
**rs2816938 (**1q32.1, *NR5A2*)				
TT	232 (83.5)	583 (88.7)	Reference	
AT+AA	46 (16.6)	74 (11.3)	1.71 (1.12–2.63)	0.01
**rs10094872** (8q24.21, *MYC*)				
AA	159 (57.2)	429 (65.3)	Reference	
TA + TT	119 (42.8)	228 (34.7)	1.50 (1.10–2.04)	0.01
**rs9581943** (13q12.2, *PDX1*)				
GG	111 (40.1)	323 (49.1)	Reference	
GA + AA	166 (59.9)	335 (50.9)	1.39 (1.03–1.88)	0.03
**rs4885093** (13q22.1, intergenic region)				
AA	59 (21.2)	196 (29.8)	Reference	
AG + GG	219 (78.8)	462 (70.2)	1.48 (1.04–2.10)	0.03

*Abbreviations CI* confidence interval, *OR* odds ratio

^a^OR and 95% CI were calculated using multivariate unconditional logistic regression model that included, age, sex, education, and the four unlinked SNPs

We conducted gene-environment analyses with four SNPs (*NR5A2* rs2816938, *MYC* rs10094872, *PDX1* rs9581943 and a chromosome 13q22 SNP, rs4885093) significantly associated with pancreatic cancer risk. Two significant (p-interaction < 0.05) gene-environment interactions were observed (Table 5). Poor oral hygiene was associated with a more significantly elevated risk of pancreatic cancer among carriers of the *NR5A2* rs2816938 AT or AA genotype (OR for 1 point increment of the oral hygiene score = 2.83, 95% CI: 1.65–4.84) compared to those with the TT genotype (OR for 1 point increment of the oral hygiene score = 1.57, 95% CI: 1.30–1.90). BMI ≧ 27 was associated with an increased pancreatic cancer risk only among individuals with the *PDX1* rs9581943 GA or AA genotype (OR = 2.99, 95% CI: 1.89–4.72) but not among those with the GG genotype (OR = 1.13, 95% CI: 0.63–2.00).

**Table 5:** The interaction between 4 genome-wide association study-identified single nucleotide polymorphisms and lifestyle and clinical factors on the risk of pancreatic cancer risk

	rs2816938 (1q32.1, *NR5A2*)		rs10094872 (8q24.21, *MYC*)		rs9581943 (13q12.2, *PDX1*)		rs4885093 (13q22.1, intergenic region)	
	TT	AT+AA	AA	TA + TT	GG	GA + AA	AA	AG + GG
	OR (95% CI)^a^	OR (95% CI)^a^	OR (95% CI)^a^	OR (95% CI)^a^	OR (95% CI)^a^	OR (95% CI)^a^	OR (95% CI)^a^	OR (95% CI)^a^
**Cigarette smoking**								
Never	Referent	Referent	Referent	Referent	Referent	Referent	Referent	Referent
Ever	1.32 (0.87–1.99)	7.41 (2.10–26.09)	1.51 (0.91–2.51)	1.92 (1.04–3.54)	2.04 (1.11–3.74)	1.39 (0.83–2.34)	0.85 (0.35–2.08)	1.91 (1.22–2.98)
	P-interaction = 0.07		P-interaction = 0.65		P-interaction = 0.73		P-interaction = 0.35	
**Oral hygiene score**^b^								
0 (Good)	Referent	Referent	Referent	Referent	Referent	Referent	Referent	Referent
1	1.51 (0.88–2.60)	2.97 (0.61–14.45)	1.65 (0.84–3.26)	1.63 (0.75–3.56)	2.07 (0.99–4.34)	1.30 (0.65–2.63)	2.06 (0.60–7.12)	1.50 (0.85–2.63)
2	2.35 (1.39–3.98)	13.26 (2.79–63.06)	2.83 (1.47–5.46)	2.93 (1.37–6.24)	2.72 (1.31–5.65)	2.71 (1.39–5.31)	3.75 (1.14–12.34)	2.55 (1.48–4.42)
3 (Poor)	3.78 (2.01–7.10)	17.64 (2.37–131.31)	4.17 (1.91–9.09)	4.74 (1.87–11.98)	4.12 (1.67–10.21)	4.20 (1.89–9.37)	4.35 (1.11–17.09)	4.37 (2.23–8.57)
	P-interaction = 0.21		P-interaction = 0.73		P-interaction = 0.66		P-interaction = 0.68	
Every 1 point increment	1.57 (1.30–1.90)	2.83 (1.65–4.84)	1.64 (1.31–2.07)	1.75 (1.33–2.31)	1.58 (1.22–2.06)	1.72 (1.35–2.19)	1.63 (1.11–2.40)	1.68 (1.38–2.05)
	P-interaction = 0.047		P-interaction = 0.71		P-interaction = 0.62		P-interaction = 0.38	
**Allergy**								
No	Referent	Referent	Referent	Referent	Referent	Referent	Referent	Referent
Yes	0.44 (0.29–0.65)	0.51 (0.21–1.23)	0.36 (0.22–0.59)	0.61 (0.36–1.05)	0.42 (0.25–0.73)	0.49 (0.30–0.79)	0.26 (0.12–0.61)	0.54 (0.36–0.81)
	P-interaction = 0.83		P-interaction = 0.16		P-interaction = 0.77		P-interaction = 0.07	
**Diabetes mellitus/glucose intolerance (DM/GI)**								
No DM/GI	Referent	Referent	Referent	Referent	Referent	Referent	Referent	Referent
< 2 years	5.61 (2.43–12.98)	1.29 (0.30–5.52)	2.67 (1.08–6.61)	8.94 (2.34–34.23)	6.20 (2.03–18.93)	2.83 (1.12–7.11)	9.11 (2.04–40.72)	3.18 (1.42–7.12)
> 2 years	3.37 (2.09–5.44)	18.50 (2.09–163.62)	3.84 (2.13–6.92)	3.48 (1.68–7.19)	4.05 (2.02–8.10)	3.27 (1.76–6.07)	4.54 (1.67–12.33)	3.70 (2.19–6.26)
	P-interaction = 0.06		P-interaction = 0.32		P-interaction = 0.46		P-interaction = 0.47	
**Vegetable consumption**								
3 portions or less per week	Referent	Referent	Referent	Referent	Referent	Referent	Referent	Referent
> 3 portions per week	0.28 (0.19–0.40)	0.41 (0.16–1.06)	0.27 (0.17–0.42)	0.34 (0.20–0.59)	0.28 (0.17–0.48)	0.31 (0.20–0.50)	0.22 (0.10–0.48)	0.31 (0.21–0.46)
	P-interaction = 0.43		P-interaction = 0.49		P-interaction = 0.71		P-interaction = 0.55	
**Fruit consumption**								
1 portion or less per day	Referent	Referent	Referent	Referent	Referent	Referent	Referent	Referent
> 1 portion per day	0.74 (0.51–1.07)	0.44 (0.16–1.21)	0.77 (0.50–1.20)	0.63 (0.36–1.08)	0.80 (0.47–1.37)	0.71 (0.45–1.11)	0.76 (0.38–1.51)	0.68 (0.46–1.01)
	P-interaction = 0.55		P-interaction = 0.56		P-interaction = 0.88		P-interaction = 0.76	
**BMI 2 years ago**^c^								
< 27	Referent	Referent	Referent	Referent	Referent	Referent	Referent	Referent
27 or more	2.21 (1.53–3.20)	1.45 (0.54–3.90)	2.00 (1.29–3.09)	2.37 (1.34–4.19)	1.13 (0.63–2.00)	2.99 (1.89–4.72)	1.48 (0.64–3.42)	2.37 (1.61–3.49)
	P-interaction = 0.46		P-interaction = 0.67		P-interaction = 0.009		P-interaction = 0.18	

*Abbreviations*: *BMI* body mass index, *CI* confidence interval, *DM/GI* diabetes mellitus/glucose intolerance, *OR* odds ratio

^a^OR and 95% CI were calculated using unconditional logistic regression, adjusted for sex, age, and education

^b^Oral hygiene score = tooth brushing + use of dental floss + regular dental visit, with tooth brushing: ≧2 times per day = 0, < 2 times per day = 1; use of dental floss: yes = 0, no = 1; and regular dental visit: yes = 0, no = 1

^c^BMI at two years before the pancreatic cancer diagnosis for the cases or before the interview date for the controls

**Fig. 1:**
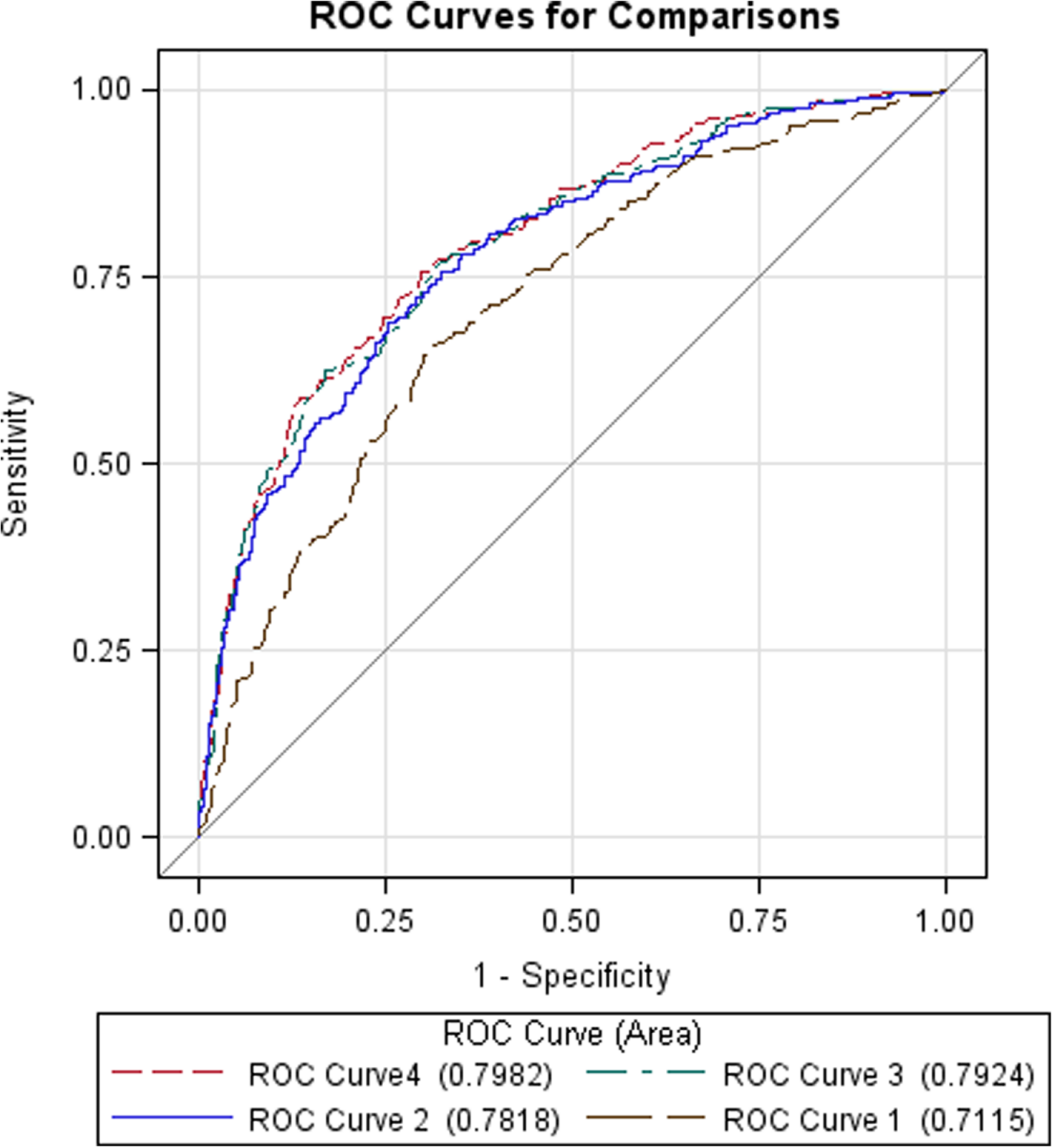
ROC curve analysis. ROC curve 1, which was constructed based on the model 1 that included age, sex, education, and the four SNPs (*NR5A2* rs2816938, *MYC* rs10094872, *PDX1* rs9581943 and a chromosome 13q22 SNPs, rs4885093), had an AUC = 0.7115. ROC curve 2, which was constructed based on the model 2 that included age, sex, education, and the seven environmental factors (cigarette smoking, oral hygiene score, vegetable consumption, fruit consumption, allergy, diabetes/glucose intolerance, and BMI) had an AUC = 0.7818, which was significantly better than the AUC of the ROC curve 1 (*p* < 0.0001). ROC curve 3, which was constructed based on the model 3 that combined the 7 environmental factors and the four SNPs plus age, sex, and education, improved the AUC to 0.7924, which was marginally better the AUC of ROC curve 2 (*p* = 0.05). ROC curve 4, which included age, sex, education, the 7 environmental factors, the four SNPs and the two significant gene-environment interactions (poor oral hygiene x *NR5A2* rs2816938 and BMI x *PDX1* rs9581943) improved the AUC further to 0.7982, which was significantly better than the AUC of ROC curve 2 (*p* = 0.006)

Fig. 1 presents the results of the ROC curve analysis. ROC curve 1, constructed with age, sex, education, and the four SNPs (*NR5A2* rs2816938, *MYC* rs10094872, *PDX1* rs9581943 and a chromosome 13q22 SNPs, rs4885093), had an AUC = 0.7115. ROC curve 2, constructed with age, sex, education, and the seven environmental factors (cigarette smoking, oral hygiene score, vegetable consumption, fruit consumption, allergy, diabetes/glucose intolerance, and BMI) had a better AUC = 0.7818 than the AUC of the ROC curve 1 (*p* < 0.0001). ROC curve 3, constructed with the 7 environmental factors and the four SNPs plus age, sex, and education, marginally improved the AUC to 0.7924 compared to the AUC of ROC curve 2 (*p* = 0.05). ROC curve 4, which included age, sex, education, the 7 environmental factors, the four SNPs and the two significant gene-environment interactions (poor oral hygiene x *NR5A2* rs2816938 and BMI x *PDX1* rs9581943) had the best AUC = 0.7982. (*p* = 0.006 when compared to the AUC of ROC curve 2).

## Discussion

Consistent with results of the previous literature, our study showed that cigarette smoking, poor dental hygiene, diabetes, and obesity were associated with an increased pancreatic cancer risk, while having allergy and more frequent consumption of vegetables and fruits were associated with a lower pancreatic cancer risk. Among the 25 pancreatic cancer GWAS-identified SNPs, 7 (rs2816938 (~ 11 kb upstream of *NR5A2*), rs10094872 (~ 28 kb upstream of *MYC*), rs9581943 (200 bp upstream of *PDX1*) and 4 chromosome 13q22.1 SNPs: rs4885093, rs9573163, rs9543325, rs9573166) showed a statistically significant association with pancreatic cancer risk. In addition, our results showed two significant gene-environment interactions (between poor oral hygiene and *NR5A2* rs2816938 and between obesity and *PDX1* rs9581943) on the risk of pancreatic cancer. The best model in differentiating pancreatic cancer cases from controls was the one that included age, sex, education, environmental factors (cigarette smoking, oral hygiene, consumption of vegetables and fruits, allergy, diabetes/glucose intolerance, and BMI), four unlinked SNPs, and the two gene-environment interactions (between poor oral hygiene and *NR5A2* rs2816938 and between obesity and *PDX1* rs9581943).

Of the 25 GWAS-identified SNPs, we were able to validate the association between 7 SNPs on four chromosomal loci (1q32.1 near *NR5A2*, 8q24.21 near *MYC*, 13q12.2 near *PDX1*, and 13q22.1) and pancreatic risk. One of the 7 SNPs, rs2816938, was previously discovered as an independent pancreatic cancer risk SNP located on a known pancreatic cancer locus, 1q32.1, by a study of subjects with European ancestry [22]. This SNP is located ~ 11 kb upstream of *NR5A2*, which encodes a transcription factor involved in the organogenesis of pancreas, including acinar differentiation [43]. Flandez et al. showed that mice with *NR5A2* heterozygosity developed a more severe acute pancreatitis with increased acino-ductal metaplasia and defective recovery from pancreatitis-induced damage [44]. In addition, *NR5A2* heterozygosity cooperated with Kras mutation in the oncogenesis of pancreatic cancer [44]. These suggested that NR5A2 could be a pancreatic tumor suppressor [44]. A second SNP (rs9581943) validated by our study was previously reported by a pancreatic cancer GWAS study with study subjects of European ancestry [28]. It is located 200 bp upstream of *PDX1*, which has been shown to regulate the initiation and the maintenance of pancreatic cancer, playing both the tumor-suppressive and oncogenic roles throughout the different stages of the pancreatic cancer development and progression [45]. Another pancreatic cancer GWAS-identified SNP (rs10094872) validated by our study was previously discovered as a pancreatic cancer risk SNP according to a study with subjects of European ancestry [22]. This SNP is located ~ 28 kb upstream of *MYC*. Previous studies have demonstrated the major contribution of *MYC* in the oncogenesis of pancreatic cancer and showed that MYC could be activated by most of the genetic and epigenetic events involved in the initiation and the progression of pancreatic cancer [46]. Four (rs4885093, rs9573163, rs9543325, and rs9573166) of the 7 SNPs validated by our study are in high LD and located on 13q22.1, which is a non-genic region previously identified as a pancreatic cancer risk locus by three studies with subjects of mostly European ancestry and a Chinese study. Besides pancreatic cancer, polymorphisms on 13q22.1 have also been associated with the risk of esophageal squamous cell carcinoma, gastric cardia cancer, and endometrial cancer [47, 48]. Functional study of this region discovered an rs386772267 (an insertion/deletion polymorphism)-containing sub-region that may regulate the expression of *DIS3* through long-range (570 kb) physical interaction [33]. More investigations are needed to decipher how this may affect the development of pancreatic cancer.

Our analysis showed a significant interaction between poor oral hygiene and *NR5A2* rs2816938 on pancreatic cancer risk. Cobo et al. showed that NR5A2 is involved in the suppression of inflammation in pancreas and a pre-inflammatory state could be observed in the pancreas of human subjects with low NR5A2 mRNA levels [49]. Poor oral hygiene may lead to the overgrowth of oral pathogenic bacteria, which have been associated with an increased pancreatic cancer risk. Michaud et al. reported that higher levels of serum antibody for *P gingivalis* ATTC53978, a periodontopathogenic bacterium, were associated with an elevated risk of pancreatic cancer [50]. By sequencing the 16S rRNA gene of the bacteria in the oral wash samples of 361 pancreatic cancer cases and 371 controls, Fan et al. found an increased risk of pancreatic cancer among individuals with carriage of oral pathogens, including *P. gingivalis* and *A. actinomycetemcomitans* [51]. Several possible biological mechanisms have been proposed to explain the association between infection with oral pathogens and pancreatic cancer. Infection with oral pathogens may generate systemic inflammation, including inflammation at a distant site, such as the pancreas, and chronic inflammation may promote carcinogenesis [52, 53]. Alternatively, oral pathogenic bacteria may travel through circulation to induce local inflammation in the pancreas to promote the oncogenesis of pancreatic cancer. The interaction between poor oral hygiene and *NR5A2* rs2816938 pointed toward the role of inflammation pathways in the oncogenesis of pancreatic cancer, although the biological mechanism underlying this interaction requires further investigation.

Other studies, although involving factors other than oral hygiene and the *NR5A2* gene, have also indicated the role of inflammation in the occurrence of pancreatic cancer. Duell et al. showed that pro-inflammatory conditions, such as pancreatitis and smoking, and pro-inflammatory genes, may have a combined effect to affect the development of pancreatic cancer [54]. Antwi et al. reported that pro-inflammatory diet, which is diet associated with higher levels of circulating inflammatory biomarkers (IL-1β, IL-4, IL-6, IL-10, TNF-α, and C-reactive protein), was associated with an increased pancreatic cancer risk [55].

Another significant gene-environment interaction in our study was between obesity and *PDX1* rs9581943. PDX1 plays an important role in maintaining the function and the survival of pancreatic beta-cells, which produce and secrete insulin [56]. A reduced expression of PDX1 may result in the pathogenesis of diabetes [56], which is a known risk factor of pancreatic cancer. Obesity is a risk factor for both pancreatic cancer and diabetes [5, 6, 57]. The interaction between obesity and *PDX1* rs9581943 in our analysis suggested the involvement of metabolic disease-related pathways in the development of pancreatic cancer.

Although to our knowledge, our study is the first to observe an interaction between obesity and *PDX1* rs9581943, polymorphisms of other genes have been reported to interact with obesity to influence pancreatic cancer risk. Tang et al. studied the polymorphisms of obesity and diabetes-associated genes and found that the associations between pancreatic cancer and the variants of *FTO* and *ADIPOQ*, which encodes the protein adiponectin, differed according to the overweight status [58]. Nakao et al. reported that the associations between the polymorphisms of *IGF1* and pancreatic cancer were significant only among the overweight individuals [59]. Studies have indicated an interplay between IGF-1 and adiponectin to influence the development of obesity, insulin resistance, diabetes, and cancer [60]. These studies again suggested the roles of metabolic disease-related pathways in the occurrence of pancreatic cancer.

This study has several limitations. First, for a hospital-based case-control study, it is difficult to determine whether the cases and the controls are from the same source population, and often the controls may not be representative of the general population. The potential selection bias of the hospital controls may have less impact for genetic association studies, unless the genetic polymorphisms were somehow associated with the hospital visits of the control subjects. Hospital controls could be unrepresentative of the general population with regards to environmental factors since they might be less healthy compared to the general population. In our control subjects, the percentage of current smokers was 21.3% for men and 3.1% for women, which were similar to the national prevalence of current smokers (26% for men and 2.3% for women) according the Annual Report of the Health Promotion Administration of Taiwan [61]. Similarly, in our control subjects, the percentage of BMI ≧ 24 (overweight + obese) 2 years before the interview date was 45.9%, which was similar to the 47.1% at the national level [61]. The comparison between our control subjects and the national data indicated that our control subjects could be representative of the general population. Second, due to the case-control study design, subjects were asked to recall exposure information in the past, such that the random recall errors might have biased the results towards the null. In addition, the case subjects might be more likely to ruminate about the possible causes of developing pancreatic cancer than the controls and this recall bias could have biased the results away from the null. Third, the sample size of the current study might not have a sufficient statistical power to validate some of the GWAS-identified SNPs, especially if the minor allele frequency is low. The minor allele frequencies of the 25 SNPs examined by the current study ranged from 0.05 to 0.50. If the minor allele frequency = 0.05 and assuming a two-sided alpha = 0.05, the current study would have a sufficient statistical power to detect a dominant OR = 1.8 and a log-additive OR = 1.75. If the minor allele frequency = 0.50 and assuming a two-sided alpha = 0.05, the current study would have a sufficient statistical power to detect a dominant OR = 1.65 and a log-additive OR = 1.33. Fourth, we did not consider adjusting for multiple testing, because all of the pancreatic cancer GWAS-identified SNPs had been well validated in the previous studies, and due to the relatively small sample size of the current study, we decided to use *p* < 0.05 as a cut-off for statistical significance. Fifth, because of the differences in genetic background and lifestyle factors, the results of the current study may not be generalizable to other ethnic/racial groups. Finally, there might be undiscovered environmental or genetic factors that could have confounded the associations observed by the current study. Further studies are needed to determine these factors.

This study has several strengths. This is the first study to validate the pancreatic cancer GWAS-identified SNPs using a case-control pancreatic cancer study conducted in Taiwan. The results indicated that some of the GWAS-identified pancreatic cancer SNPs may also contribute to the pancreatic cancer occurring in Taiwan but the non-replications of the majority of the 25 SNPs also indicated that there may be pancreatic cancer-associated genetic polymorphisms that are specific to the Taiwanese population and further investigations are required. Another strength of this study is the investigation of gene-environment interaction. We were able to show the significant interaction between poor oral hygiene and *NR5A2* rs2816938 and between obesity and *PDX1* rs9581943, which suggested the involvement of inflammation and metabolic disease-related pathways in the development of pancreatic cancer.

## Conclusions

Our study confirmed the association between 7 GWAS-identified SNPs and pancreatic risk among the Taiwanese population. Our results also showed that pancreatic cancer was jointly influenced by lifestyle and medical factors, genetic polymorphisms, and gene-environment interaction. Some of the GWAS-identified SNPs were not validated by our study, which might be explained partly by racial/ethnic differences in genetic background. In the future, we plan to conduct a GWAS of pancreatic cancer in Taiwan to determine additional genetic polymorphisms that are more relevant to the pancreatic cancer cases occurring in Taiwan.

## Supplementary information

**Additional file 1: Table S1.** Descriptions of the 25 genome-wide association study-identified single nucleotide polymorphisms of pancreatic cancer

**Additional file 2: Table S2.** The association between 25 genome-wide association study-identified single nucleotide polymorphisms and the risk of pancreatic cancer

## Data Availability

All data generated during this study are included in this published article.

## Abbreviations

AUC: Area under the curve
BMI: Body mass index
CI: Confidence interval
GWAS: Genome-wide association studies
IARC: International Agency for Research on Cancer
LD: Linkage disequilibrium
OR: Odds ratio
ROC: Receiver operating characteristic
SNP: Single nucleotide polymorphism
