# Supplementary material for: Validation of genome-wide association study-identified single nucleotide polymorphisms in a case-control study of pancreatic cancer from Taiwan
Source: J Biomed Sci. 2020 May 26;27:69. doi: 10.1186/s12929-020-00664-9 (PMC7251895; doi:10.1186/s12929-020-00664-9)
Supplement: Supplementary file 1 — Additional file 1: Table S1. Descriptions of the 25 genome-wide association study-identified single nucleotide polymorphisms of pancreatic cancer [file 12929_2020_664_MOESM1_ESM.doc]

Table S1. Descriptions of the 25 genome-wide association study-identified single nucleotide polymorphisms of pancreatic cancer

| Single nucleotide polymorphism | Chromosome locus | Nearest gene(s) | Major/minor alleles | References | Race/ethnic group of the study population | OR (95% CI) associated with each copy of minor allele |
| --- | --- | --- | --- | --- | --- | --- |
| rs2816938 | 1q32.1 | *NR5A2* | T/A | Zhang et al. | European ancestry | 1.20 (1.15-1.25) |
| rs3790843 | 1q32.1 | *NR5A2* | G/A | Petersen et al. | Mostly European ancestry | 0.81 (0.75-0.87) |
|  |  |  |  | Ueno et al. | Japanese | 0.78 (0.62-0.99) |
| rs3790844 | 1q32.1 | *NR5A2* | T/C | Petersen et al. | Mostly European ancestry | 0.77 (0.71-0.84) |
|  |  |  |  | Ueno et al. | Japanese | 0.70 (0.56-0.89) |
| rs1486134 | 2p13.3 | *ETAA1* | T/G | Childs et al. | >92% with European ancestry | 1.14 (1.09-1.19) |
| rs9854771 | 3q29 | *TP63* | G/A | Childs et al. | >92% with European ancestry | 0.89 (0.85-0.93) |
| rs2255280 | 5p13.1 | *DAB2* | T/G | Wu et al. | Chinese | 0.81 (0.76-0.87) |
| rs36115365 | 5p15.33 | *TERT, CLPTM1L* | G/C | Fang et al. | Mostly European ancestry | 1.29 (1.23-1.34) |
| rs2736098 | 5p15.33 | *TERT*  *CLPTM1L* | C/T | Wolpin et al. | European ancestry | 0.80 (0.76-0.85) |
| rs401681 | 5p15.33 | *TERT, CLPTM1L* | C/T | Petersen et al. | Mostly European ancestry | 1.19 (1.11-1.27) |
|  |  |  |  | Liu et al. | Chinese | 1.20 (1.06-1.44) |
| rs9502893 | 6p25.3 | *FOXQ1* | A/G | Low et al. | Japanese | 1.29 (1.17-1.43) |
| rs10094872 | 8q24.21 | *MYC* | A/T | Zhang et al. | European ancestry | 1.15 (1.10-1.20) |
| rs505922 | 9q34.2 | *ABO* | T/C | Amundadottir et al | Mostly European ancestry | 1.20 (1.12-1.28) |
|  |  |  |  | Willis et al. | 93% Caucasians | 1.65 (1.20-2.26) |
| rs4962153 | 9q34.2 | *ADAMTS13*, *ABO* | G/A | Wolpin et al | European ancestry | 1.20 (1.10-1.30) |
| rs12413624 | 10q26.11 | *PRLHR* | A/T | Wu et al. | Chinese | 1.23 (1.16-1.31) |
| rs708224 | 12p11 | *BICD1* | A/G | Low et al. | Japanese | 1.32 (1.19-1.47) (reported for allele A) |
| rs9581943 | 13q12.2 | *PDX1* | G/A | Wolpin et al. | European ancestry | 1.15 (1.10-1.20) |
| rs4885093 | 13q22.1 | None | T/C | Wu et al. | Chinese | 1.25 (1.18-1.33) |
| rs9573163 | 13q22.1 | None | C/G | Wu et al. | Chinese | 1.26 (1.18-1.34) |
| rs9543325 | 13q22.1 | None | T/C | Petersen et al. | Mostly European ancestry | 1.26 (1.18-1.35) |
|  |  |  |  | Willis et al. | 93% Caucasians | 1.42 (1.06-1.90) |
| rs9573166 | 13q22.1 | None | A/G | Hoskins et al. | Mostly European Ancestry | 1.25 (1.18-1.32) |
| rs11655237 | 17q25.1 | *LINC00673* | C/T | Childs et al. | >92% with European ancestry | 1.26 (1.19-1.34) |
| rs372883 | 21q21.3 | *BACH1* | T/C | Wu et al. | Chinese | 0.79 (0.75-0.84) |
| rs1547374 | 21q22.3 | *TFF1* | A/G | Wu et al. | Chinese | 0.79 (0.74-0.84) |
| rs16986825 | 22q12.1 | *ZNRF3* | C/T | Wolpin et al. | European ancestry | 1.18 (1.12-1.25) |
| rs5768709 | 22q13.32 | *FAM19A5* | A/G | Wu et al. | Chinese | 1.25 (1.17-1.34) |

Abbreviations: CI: confidence interval; OR: odds ratio

**References**

1. Zhang M, Wang Z, Obazee O, Jia J, Childs EJ, Hoskins J, et al. Three new pancreatic cancer susceptibility signals identified on chromosomes 1q32.1, 5p15.33 and 8q24.21. Oncotarget. 2016;7:66328-43.

2. Petersen GM, Amundadottir L, Fuchs CS, Kraft P, Stolzenberg-Solomon RZ, Jacobs KB, et al. A genome-wide association study identifies pancreatic cancer susceptibility loci on chromosomes 13q22.1, 1q32.1 and 5p15.33. Nat Genet. 2010;42:224-8.

3. Ueno M, Ohkawa S, Morimoto M, Ishii H, Matsuyama M, Kuruma S, et al. Genome-wide association study-identified SNPs (rs3790844, rs3790843) in the NR5A2 gene and risk of pancreatic cancer in Japanese. Sci Rep. 2015;5:17018.

4. Childs EJ, Mocci E, Campa D, Bracci PM, Gallinger S, Goggins M, et al. Common variation at 2p13.3, 3q29, 7p13 and 17q25.1 associated with susceptibility to pancreatic cancer. Nat Genet. 2015;47:911-6.

5. Wu C, Miao X, Huang L, Che X, Jiang G, Yu D, et al. Genome-wide association study identifies five loci associated with susceptibility to pancreatic cancer in Chinese populations. Nat Genet. 2011;44:62-6.

6. Fang J, Jia J, Makowski M, Xu M, Wang Z, Zhang T, et al. Functional characterization of a multi-cancer risk locus on chr5p15.33 reveals regulation of TERT by ZNF148. Nat Commun. 2017;8:15034.

7. Wolpin BM, Rizzato C, Kraft P, Kooperberg C, Petersen GM, Wang Z, et al. Genome-wide association study identifies multiple susceptibility loci for pancreatic cancer. Nat Genet. 2014;46:994-1000.

8. Liu C, Wang Y, Huang H, Wang C, Zhang H, Kong Y, et al. Association between CLPTM1L-TERT rs401681 polymorphism and pancreatic cancer risk among Chinese Han population. Tumour Biol. 2014;35:5453-7.

9. Low SK, Kuchiba A, Zembutsu H, Saito A, Takahashi A, Kubo M, et al. Genome-wide association study of pancreatic cancer in Japanese population. PLoS One. 2010;5:e11824.

10. Amundadottir L, Kraft P, Stolzenberg-Solomon RZ, Fuchs CS, Petersen GM, Arslan AA, et al. Genome-wide association study identifies variants in the ABO locus associated with susceptibility to pancreatic cancer. Nat Genet. 2009;41:986-90.

11. Willis JA, Olson SH, Orlow I, Mukherjee S, McWilliams RR, Kurtz RC, et al. A replication study and genome-wide scan of single-nucleotide polymorphisms associated with pancreatic cancer risk and overall survival. Clin Cancer Res. 2012;18:3942-51.

12. Hoskins JW, Ibrahim A, Emmanuel MA, Manmiller SM, Wu Y, O'Neill M, et al. Functional characterization of a chr13q22.1 pancreatic cancer risk locus reveals long-range interaction and allele-specific effects on DIS3 expression. Hum Mol Genet. 2016;25:4726-38.
