# Supplementary material for: Validation of genome-wide association study-identified single nucleotide polymorphisms in a case-control study of pancreatic cancer from Taiwan
Source: J Biomed Sci. 2020 May 26;27:69. doi: 10.1186/s12929-020-00664-9 (PMC7251895; doi:10.1186/s12929-020-00664-9)
Supplement: Supplementary file 2 — Additional file 2: Table S2. The association between 25 genome-wide association study-identified single nucleotide polymorphisms and the risk of pancreatic cancer [file 12929_2020_664_MOESM2_ESM.doc]

Table S2. The association between 25 genome-wide association study-identified single nucleotide polymorphisms and the risk of pancreatic cancer

| **SNPs (chromosome locus, nearest gene (s))** | **Cases**  **n (%)** | **Controls**  **n (%)** | OR (95% CI)^a^ | P |
| --- | --- | --- | --- | --- |
| **rs2816938 (**1q32.1, *NR5A2*) |  |  |  |  |
| TT | 232 (83.5) | 583 (88.7) | Reference |  |
| AT | 44 (15.8) | 72 (11.0) | 1.68 (1.09-2.58) | 0.02 |
| AA | 2 (0.7) | 2 (0.3) | 1.58 (0.22-11.39) | 0.65 |
| AT+AA | 46 (16.6) | 74 (11.3) | 1.68 (1.10-2.55) | 0.02 |
| Every 1 copy of A |  |  | 1.61 (1.08-2.40) | 0.02 |
| **rs3790843** (1q32.1, *NR5A2*) |  |  |  |  |
| TT | 131 (47.1) | 340 (51.7) | Reference |  |
| TC | 124 (44.6) | 275 (41.8) | 1.14 (0.84-1.55) | 0.40 |
| CC | 23 (8.3) | 43 (6.5) | 1.54 (0.87-2.72) | 0.14 |
| TC+CC | 147 (52.9) | 318 (48.3) | 1.19 (0.89-1.60) | 0.24 |
| Every 1 copy of C |  |  | 1.19 (0.94-1.51) | 0.14 |
| **rs3790844** (1q32.1, *NR5A2*) |  |  |  |  |
| GG | 116 (41.7) | 295 (44.8) | Reference |  |
| GA | 131 (47.1) | 297 (45.1) | 1.12 (0.82-1.53) | 0.47 |
| AA | 31 (11.2) | 66 (10.0) | 1.31 (0.79-2.17) | 0.29 |
| GA+AA | 162 (58.3) | 363 (55.2) | 1.16 (0.86-1.56) | 0.34 |
| Every 1 copy of A |  |  | 1.14 (0.91-1.42) | 0.26 |
| **rs1486134** (2p13.3, *ETAA1*) |  |  |  |  |
| TT | 81 (29.1) | 224 (34.1) | Reference |  |
| GT | 148 (53.3) | 303 (46.2) | 1.21 (0.86-1.69) | 0.28 |
| GG | 49 (17.6) | 129 (19.7) | 1.06 (0.69-1.64) | 0.79 |
| GT+GG | 197 (70.9) | 432 (65.9) | 1.16 (0.84-1.60) | 0.36 |
| Every 1 copy of G |  |  | 1.05 (0.85-1.30) | 0.65 |
| **rs9854771** (3q29, *TP63*) |  |  |  |  |
| GG | 197 (70.9) | 465 (70.8) | Reference |  |
| AG | 76 (27.3) | 176 (26.8) | 1.03 (0.74-1.44) | 0.84 |
| AA | 5 (1.8) | 16 (2.4) | 0.66 (0.22-1.92) | 0.44 |
| AG+AA | 81 (29.1) | 192 (29.2) | 1.00 (0.72-1.38) | 1.00 |
| Every 1 copy of A |  |  | 0.97 (0.72-1.29) | 0.82 |
| **rs2255280** (5p13.1, *DAB2*) |  |  |  |  |
| AA | 99 (35.6) | 244 (37.2) | Reference |  |
| CA | 133 (47.8) | 308 (15.8) | 1.11 (0.80-1.53) | 0.54 |
| CC | 46 (16.6) | 104 (47.0) | 1.20 (0.77-1.86) | 0.42 |
| CA+ CC | 179 (64.4) | 412 (62.8) | 1.13 (0.83-1.54) | 0.44 |
| Every 1 copy of C |  |  | 1.10 (0.89-1.36) | 0.39 |
| **rs36115365** (5p15.33, *TERT, CLPTM1L*) |  |  |  |  |
| GG | 195 (70.1) | 492 (74.8) | Reference |  |
| GC | 77 (27.7) | 155 (23.6) | 1.28 (0.91-1.78) | 0.16 |
| CC | 6 (2.2) | 11 (1.7) | 1.36 (0.47-3.91) | 0.57 |
| GC+CC | 83 (29.9) | 166 (25.3) | 1.28 (0.92-1.78) | 0.14 |
| Every 1 copy of C |  |  | 1.24 (0.93-1.67) | 0.14 |
| **rs2736098** (5p15.33, *TERT, CLPTM1L*) |  |  |  |  |
| CC | 113 (40.7) | 280 (42.6) | Reference |  |
| TC | 134 (11.1) | 294 (12.6) | 1.18 (0.86-1.61) | 0.31 |
| TT | 31 (48.2) | 83 (44.8) | 0.96 (0.59-1.56) | 0.85 |
| TC+TT | 165 (50.3) | 377 (57.4) | 1.13 (0.84-1.52) | 0.43 |
| Every 1 copy of T |  |  | 1.03 (0.83-1.29) | 0.76 |
| **rs401681** (5p15.33, *TERT, CLPTM1L*) |  |  |  |  |
| CC | 116 (41.9) | 315 (47.9) | Reference |  |
| CT | 127 (45.8) | 283 (43.0) | 1.18 (0.86-1.61) | 0.31 |
| TT | 34 (12.3) | 60 (9.1) | 1.40 (0.86-2.31) | 0.18 |
| CT+TT | 161 (58.1) | 343 (52.1) | 1.22 (0.90-1.64) | 0.20 |
| Every 1 copy of T |  |  | 1.18 (0.95-1.48) | 0.14 |
| **rs9502893** (6p25.3, *FOXQ1*) |  |  |  |  |
| TT | 97 (34.9) | 233 (35.4) | Reference |  |
| CT | 140 (50.4) | 310 (47.1) | 1.13 (0.82-1.56) | 0.47 |
| CC | 41 (14.7) | 115 (17.5) | 0.92 (0.59-1.44) | 0.70 |
| CT+CC | 181 (65.1) | 425 (64.6) | 1.07 (0.79-1.46) | 0.66 |
| Every 1 copy of C |  |  | 0.99 (0.80-1.22) | 0.92 |
| **rs10094872** (8q24.21, *MYC*) |  |  |  |  |
| AA | 159 (57.2) | 429 (65.3) | Reference |  |
| TA | 111 (39.9) | 196 (29.8) | 1.61 (1.19-2.22) | 0.002 |
| TT | 8 (2.9) | 32 (4.9) | 0.62 (0.27-1.44) | 0.27 |
| TA+TT | 119 (42.8) | 228 (34.7) | 1.48 (1.09-2.00) | 0.01 |
| Every 1 copy of T |  |  | 1.23 (0.95-1.59) | 0.11 |
| **rs505922** (9q34.2, *ABO*) |  |  |  |  |
| TT | 112 (40.6) | 280 (42.7) | Reference |  |
| CT | 128 (46.4) | 292 (44.6) | 1.05 (0.76-1.44) | 0.77 |
| CC | 36 (13.0) | 83 (12.7) | 0.97 (0.61-1.55) | 0.90 |
| CC+CC | 164 (59.4) | 375 (57.3) | 1.03 (0.76-1.39) | 0.85 |
| Every 1 copy of C |  |  | 1.00 (0.81-1.24) | 0.99 |
| **rs4962153** (9q34.2, *ADAMTS13, ABO*) |  |  |  |  |
| GG | 248 (89.2) | 587 (89.4) | Reference |  |
| AG | 28 (10.1) | 68 (10.3) | 0.95 (0.58-1.55) | 0.84 |
| AA | 2 (0.7) | 2 (0.3) | 2.42 (0.33-17.96) | 0.38 |
| AG+AA | 30 (10.8) | 70 (10.6) | 1.00 (0.62-1.60) | 0.98 |
| Every 1 copy of A |  |  | 1.04 (0.67-1.62) | 0.87 |
| **rs12413624** (10q26.11, *PRLHR*) |  |  |  |  |
| TT | 113 (40.6) | 283 (43.0) | Reference |  |
| TA | 137 (49.3) | 297 (45.1) | 1.14 (0.84-1.56) | 0.40 |
| AA | 28 (10.1) | 78 (11.9) | 0.97 (0.59-1.62) | 0.92 |
| TA+AA | 165 (59.4) | 375 (57.0) | 1.11 (0.82-1.50) | 0.49 |
| Every 1 copy of A |  |  | 1.04 (0.83-1.30) | 0.74 |
| **rs708224** (12p11, *BICD1*) |  |  |  |  |
| AA | 77 (27.8) | 165 (25.2) | Reference |  |
| AG | 139 (50.2) | 330 (50.3) | 0.92 (0.65-1.31) | 0.64 |
| GG | 61 (22.0) | 161 (24.5) | 0.83 (0.54-1.26) | 0.38 |
| AG+GG | 200 (72.2) | 491 (74.8) | 0.89 (0.64-1.24) | 0.49 |
| Every 1 copy of G |  |  | 0.91 (0.74-1.12) | 0.38 |
| **rs9581943** (13q12.2, *PDX1*) |  |  |  |  |
| GG | 111 (40.1) | 323 (49.1) | Reference |  |
| GA | 126 (45.5) | 250 (38.0) | 1.41 (1.03-1.94) | 0.03 |
| AA | 40 (14.4) | 85 (12.9) | 1.38 (0.87-2.17) | 0.17 |
| GA+AA | 166 (59.9) | 335 (50.9) | 1.40 (1.04-1.89) | 0.03 |
| Every 1 copy of A |  |  | 1.23 (1.00-1.52) | 0.05 |
| **rs4885093** (13q22.1, intergenic region) |  |  |  |  |
| AA | 59 (21.2) | 196 (29.8) | Reference |  |
| AG | 150 (54.0) | 335 (50.9) | 1.43 (0.99-2.06) | 0.06 |
| GG | 69 (24.8) | 127 (19.3) | 1.65 (1.07-2.54) | 0.02 |
| AG+GG | 219 (78.8) | 462 (70.2) | 1.49 (1.05-2.11) | 0.02 |
| Every 1 copy of G |  |  | 1.29 (1.04-1.59) | 0.02 |
| **rs9573163** (13q22.1, intergenic region) |  |  |  |  |
| GG | 59 (21.2) | 195 (29.6) | Reference |  |
| GC | 150 (54.0) | 337 (51.2) | 1.40 (0.97-2.02) | 0.07 |
| CC | 69 (24.8) | 126 (19.2) | 1.64 (1.06-2.53) | 0.03 |
| GC+CC | 219 (78.8) | 463 (70.4) | 1.47 (1.04-2.08) | 0.03 |
| Every 1 copy of C |  |  | 1.28 (1.03-1.59) | 0.02 |
| **rs9543325** (13q22.1, intergenic region) |  |  |  |  |
| TT | 62 (22.3) | 198 (30.1) | Reference |  |
| TC | 146 (52.5) | 333 (50.7) | 1.40 (0.99-1.98) | 0.06 |
| CC | 70 (25.2) | 126 (19.2) | 1.77 (1.18-2.67) | 0.006 |
| TC+CC | 216 (77.7) | 459 (69.9) | 1.44 (1.02-2.02) | 0.04 |
| Every 1 copy of C |  |  | 1.28 (1.03-1.58) | 0.02 |
| **rs9573166** (13q22.1, intergenic region) |  |  |  |  |
| AA | 62 (22.4) | 193 (29.4) | Reference |  |
| GA | 145 (52.3) | 335 (51.0) | 1.29 (0.90-1.86) | 0.17 |
| GG | 70 (25.3) | 129 (19.6) | 1.56 (1.02-2.39) | 0.04 |
| GA+GG | 215 (77.6) | 464 (70.6) | 1.37 (0.97-1.93) | 0.08 |
| Every 1 copy of G |  |  | 1.25 (1.01-1.55) | 0.04 |
| **rs11655237** (17q25.1, *LINC00673*) |  |  |  |  |
| CC | 173 (62.2) | 416 (63.2) | Reference |  |
| CT | 93 (33.5) | 215 (32.7) | 1.02 (0.74-1.40) | 0.90 |
| TT | 12 (4.3) | 27 (4.1) | 1.02 (0.49-2.14) | 0.95 |
| CT+TT | 105 (37.8) | 242 (36.8) | 1.02 (0.75-1.39) | 0.89 |
| Every 1 copy of T |  |  | 1.02 (0.79-1.32) | 0.90 |
| **rs372883** (21q21.3, *BACH1*) |  |  |  |  |
| TT | 80 (28.8) | 171 (26.0) | Reference |  |
| TC | 145 (52.2) | 335 (50.9) | 0.95 (0.67-1.34) | 0.77 |
| CC | 53 (19.0) | 152 (23.1) | 0.76 (0.49-1.17) | 0.21 |
| TC+CC | 198 (71.2) | 487 (74.0) | 0.89 (0.64-1.24) | 0.48 |
| Every 1 copy of C |  |  | 0.88 (0.71-1.08) | 0.22 |
| **rs1547374** (21q22.3, *TFF1*) |  |  |  |  |
| AA | 83 (29.9) | 202 (30.7) | Reference |  |
| AG | 142 (51.0) | 309 (47.0) | 1.12 (0.80-1.57) | 0.52 |
| GG | 53 (19.1) | 147 (22.3) | 0.92 (0.60-1.40) | 0.69 |
| AG+GG | 195 (70.1) | 456 (69.3) | 1.06 (0.77-1.45) | 0.74 |
| Every 1 copy of G |  |  | 0.97 (0.79-1.20) | 0.80 |
| **rs16986825** (22q12.1, *ZNRF3*) |  |  |  |  |
| CC | 84 (30.3) | 218 (33.1) | Reference |  |
| TC | 137 (49.5) | 329 (50.0) | 1.05 (0.75-1.47) | 0.79 |
| TT | 56 (20.2) | 111 (16.9) | 1.31 (0.85-2.01) | 0.22 |
| TC+TT | 193 (69.7) | 440 (66.9) | 1.11 (0.81-1.53) | 0.52 |
| Every 1 copy of T |  |  | 1.13 (0.91-1.40) | 0.26 |
| **rs5768709** (22q13.32, *FAM19A5*) |  |  |  |  |
| AA | 161 (58.1) | 394 (59.9) | Reference |  |
| AG | 107 (38.6) | 231 (35.1) | 1.20 (0.88-1.63) | 0.26 |
| GG | 9 (3.3) | 33 (5.0) | 0.74 (0.34-1.63) | 0.45 |
| AG+GG | 116 (41.9) | 264 (40.1) | 1.14 (0.85-1.54) | 0.39 |
| Every 1 copy of G |  |  | 1.05 (0.82-1.36) | 0.69 |

Abbreviations: CI: confidence interval; OR: odds ratio

a. OR and 95% CI were calculated using unconditional logistic regression, adjusted for age, sex, and education
